# Pathophysiological Role and Therapeutic Implications of Vitamin D in Autoimmunity: Focus on Chronic Autoimmune Diseases

**DOI:** 10.3390/nu12030789

**Published:** 2020-03-17

**Authors:** Mattia Bellan, Laura Andreoli, Chiara Mele, Pier Paolo Sainaghi, Cristina Rigamonti, Silvia Piantoni, Carla De Benedittis, Gianluca Aimaretti, Mario Pirisi, Paolo Marzullo

**Affiliations:** 1 Department of Translational Medicine, Università del Piemonte Orientale UPO, 28100 Novara, Italy; mattia.bellan@med.uniupo.it (M.B.); chiara.mele1989@gmail.com (C.M.); pierpaolo.sainaghi@med.uniupo.it (P.P.S.); cristina.rigamonti@med.uniupo.it (C.R.); karla-89@hotmail.it (C.D.B.); gianluca.aimaretti@med.uniupo.it (G.A.); mario.pirisi@med.uniupo.it (M.P.); 2 Division of Internal Medicine, “AOU Maggiore della Carità”, 28100 Novara, Italy; 3 CAAD, Centre for Autoimmune and Allergic Diseases, 28100 Novara, Italy; 4 Rheumatology and Clinical Immunology Unit and Department of Clinical and Experimental Sciences, Spedali Civili and University of Brescia, 25128 Brescia, Italy; laura.andreoli@unibs.it (L.A.); slv.piantoni@gmail.com (S.P.); 5 Division of General Medicine, Ospedale S. Giuseppe, I.R.C.C.S. Istituto Auxologico Italiano, 28921 Verbania, Italy

**Keywords:** vitamin D, autoimmunity, autoimmune diseases, rheumatoid arthritis, spondyloarthritis, systemic lupus erythematosus, antiphospholipid syndrome, type 1 diabetes mellitus, autoimmune thyroid disease, Addison’s disease, autoimmune liver disease

## Abstract

Vitamin D is a pleiotropic secosteroid yielding multiple actions in human physiology. Besides the canonical regulatory activity on bone metabolism, several non-classical actions have been described and the ability of vitamin D to partake in the regulation of the immune system is particularly interesting, though far stronger and convincing evidence has been collected in in vitro as compared to in vivo studies. Whether vitamin D is able to regulate at physiological concentrations the human immune system remains unproven to date. Consequently, it is not established if vitamin D status is a factor involved in the pathogenesis of immune-mediated diseases and if cholecalciferol supplementation acts as an adjuvant for autoimmune diseases. The development of autoimmunity is a heterogeneous process, which may involve different organs and systems with a wide range of clinical implications. In the present paper, we reviewed the current evidences regarding vitamin D role in the pathogenesis and management of different autoimmune diseases.

## 1. Introduction

Vitamin D is a hormone, firstly characterized for its prominent role in bone homeostasis: in the early decades of the twentieth century, the discovery that rickets might be prevented and treated by sun exposure led to isolating and identifying vitamin D2 (ergocalciferol) and vitamin D3 (cholecalciferol) [1]. In the following decades, our knowledge about vitamin D physiology grew significantly; indeed, vitamin D activity is integrated in a complex hormonal axis along with parathyroid hormone (PTH) and fibroblast growth factor 23 [2], such that cholecalciferol acts to spare calcium at the level of the gut, the bone, the parathyroid glands and the kidney [3,4]. Besides the increasing awareness of the complexity of vitamin D activity on bone, the isolation of vitamin D receptor (VDR) in many different cell types made evident the wider role of this hormone in human physiology. Thus, more and more activities have been attributed to vitamin D: it contributes to the development of the nervous system, as well as to its protection, transmission, and plasticity [5]. Moreover, vitamin D exerts a protective role on the vascular endothelium [6,7,8], downregulates the renin-angiotensin-aldosterone system [9], and positively regulates insulin sensitivity [10], making vitamin D status a putative determinant of atherogenesis and a potential novel biomarker for cardiovascular risk [11]. Vitamin D status is also considered as a marker of general health, since low vitamin D levels are associated with cardio-metabolic disorders, neuromuscular disorders, cancer, and longevity [12].

Importantly, evidence has accumulated on the complex regulatory role of Vitamin D on the immune system, which is the result of its actions on both innate and adaptive immunity. Vitamin D contributes to hematopoiesis, since VDR drives the myeloid differentiation towards monocytes and granulocytes [13], and is involved in antimicrobial response, particularly against Mycobacteria infections. The exposure of human monocytes to such pathogens upregulates VDR expression. Further, the local activation of vitamin D is enhanced by the upregulation of the activating enzyme CYP27B1; a clinically relevant implication of this process is the increased risk of hypercalcemia which is observed in the context of granulomatous diseases, as a result of excessive local activation of cholecalciferol, which is released from the classical mechanisms of negative feedback [14]. 

**Figure 1:**
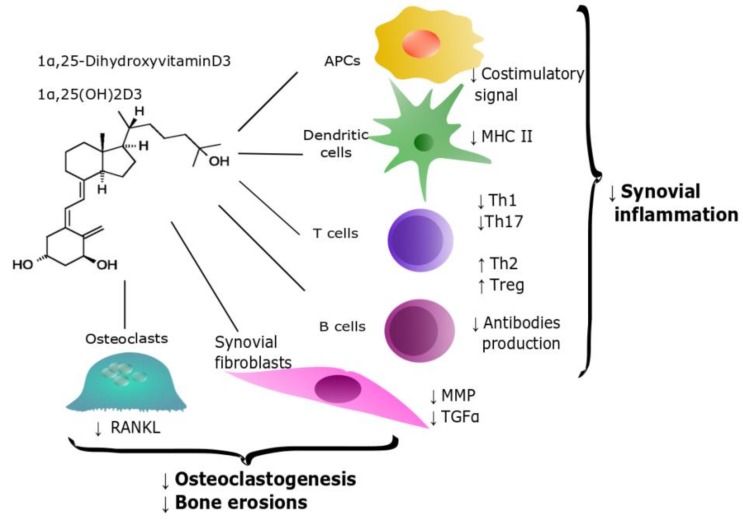
Scheme of 1α,25(OH)2D3 role in regulating immune response. As reviewed, it interacts both with innate- and adaptive-immune cells and with resident synoviocytes as well as osteoclasts, resulting in a decrease of synovial inflammation and, finally, in bone erosion. Arrows are used to illustrate decreased (↓) or increased (↑) production of specific actions, cells or molecules.

The active form of vitamin D, 1,25(OH)2D3 (also called calcitriol) increases antimicrobial activity of macrophages and monocytes by enhancing the production of cathelicidin antimicrobial peptide (CAMP) and defensin β2 [15,16]. Moreover, 1,25(OH)2D3 induces chemotaxis and autophagy of innate immune cells [17,18] and upregulates their phagocytic activity, contributing to the clearance of pathogens [19]. In addition, vitamin D prevents infections by restricting the spreading of pathogens, via neutrophil extracellular traps (NETs) formation, as recently proposed [20]. A schematic illustration of 1α,25(OH)2D3 role in regulating the immune response is depicted in Figure 1.

While on the one hand the active form of vitamin D promotes the antimicrobial activity of innate immunity, on the other it downregulates antigen presentation by monocytes, probably contributing to immune tolerance [21,22]. Additionally, 1,25(OH)2D3 drives monocytes differentiation towards macrophages [23] instead of dendritic cells (DCs) [24]. More generally, DCs activity is also impaired by 1,25(OH)2D3, which inhibits DCs chemotaxis and antigen presentation, by downregulating MHC II and costimulatory molecules expression [25,26]. 

The immunomodulatory effects of vitamin D on adaptive immunity are both indirect and direct. First of all, the spreading of adaptive response is negatively affected by the suppressive effect on antigen presentation. Moreover, vitamin D is able to downregulate the expression of proinflammatory cytokines by monocytes, such as Interleukin 6 (IL-6) and Tumor Necrosis Factor α (TNFα), which are part of the inflammatory milieu allowing B and T cells activation and proliferation [27]. 

VDR is expressed by B cells at low levels in quiescence and it is induced upon B cells activation [28]. The effects of 1,25(OH)2D3 on B cells are multiple: calcitriol induces B cells apoptosis, thus preventing proliferation and differentiation into plasma cells. By doing it, early administration of 1,25(OH)2D3 to stimulated B cells cultures reduces immunoglobulin secretion [29]. Furthermore, the in vitro activity of B cells as antigen presenting cells (APC) is inhibited by vitamin D, possibly through inhibition of the costimulatory signal mediated by CD86 [30]. 

T cells are another target of vitamin D action. 1,25(OH)2D3 inhibits the cytotoxic activity of T cells by suppressing the expression of Fas-ligand [31]. Vitamin D also has many effects on T helper cells (Th). Calcitriol drives CD4+ Th differentiation, leading to a reduction of Th1 and Th17 cells [32]. Th1 and Th17 subsets elicit a key role in different chronic inflammatory diseases, driving the inflammatory response by the release of cytokines. It is not surprising that the exposure to 1,25(OH)2D3 diminishes the expression of Th1 (IL-2, TNF-α, and Interferon γ- IFNγ) as well as of cytokines associated with Th17 (IL-17A, IL-17F, IL-21, IL-22) [33,34]. Conversely, 1,25(OH)2D3 polarizes CD4+ cells towards a Th2 phenotype with upregulation of cytokines such as IL-4 and IL-5 [35,36]. Finally, 1,25(OH)2D3 induces the differentiation of Treg, CD4+ cells involved in the maintenance of immune tolerance, by enhancing the expression of CTLA-4 and Foxp3. This in turn leads to an increase of IL-10 and transforming growth factor β1 (TGF-β1) [37,38]. 

In summary, there is strong evidence originating from studies showing that vitamin D is, at least in vitro, a regulator of multiple subsets of the immune system. However, the relevance of vitamin D-mediated regulation of the immune system in vivo is still to be proven. More specifically, it is unclear whether these potential beneficial effects are exerted at the plasma concentrations normally achieved in vivo or, conversely, if higher levels are required. 

In this paper, our aim was to review the current evidences linking vitamin D to the regulation of immune system activity, specifically focusing on different autoimmune diseases.

## 2. Vitamin D and Inflammatory Arthritis 

### 2.1. Rheumatoid Arthritis

Rheumatoid Arthritis (RA) is the most prevalent rheumatic chronic inflammatory disease worldwide; though characterized by the development of peripheral synovitis, it is considered a systemic disease linked to genetic and environmental factors. Looking at the effects that vitamin D plays on the immune system, different groups evaluated the potential pathogenetic relevance of vitamin D status on the development of RA. Moreover, despite the groundbreaking revolution caused by the introduction of biologics in the management of the disease, which dramatically improved its clinical and functional prognosis, there is still an ongoing search for novel drugs. In this context, vitamin D has some interest.

Since the late years of last century, different groups reported a potential protective and therapeutic effect of vitamin D on murine models of arthritis. The relevance of vitamin D in the pathogenesis of RA is supported by the observation that VDR-deficient mice are prone to a more severe arthritis when crossed with human TNF transgenic mice (hTNFtg), in comparison to VDR wild type/hTNFtg mice. VDR-deficient mice showed larger synovial infiltrates of inflammatory cells, and their monocytes demonstrated a pro-inflammatory phenotype, being particularly prone to differentiate into osteoclasts [39]. Tsuji et al., in 1994, reported that the oral administration of 1,25(OH)2D3 is able to protect mice from the development of collagen induced arthritis (CIA) caused by the administration of type II collagen (CII) [40]. Similarly, the non-hypercalcemic analogue MC 1288 (20-epi-1α,25-dihydroxycholecalciferol) prevents CIA development when administered before CII immunization; indeed, when started in mice with established CIA, MC 1288 is able to reduce the severity of arthritis [41]. In a further model of RA (Adjuvant-induced arthritis, ATA), the effect of vitamin D status on disease course was tested. Rats fed with a vitamin D-replete diet showed less severe arthritis than those fed with a vitamin D deficient diet [42]. These observations suggest that, in animal models, vitamin D signaling protects from arthritis and might be exploited for treatment. 

Preclinical data suggest that 1,25(OH)2D3 can effectively regulate monocytes activity, promoting an anti-inflammatory phenotype. When primary cultures of macrophages derived from peripheral blood monocyte obtained from patients with RA are treated with 1,25(OH)2D3, TNF-α and receptor activator of nuclear factor-κB ligand (RANKL) production dose-dependently decrease. Moreover, at higher concentrations, 1,25(OH)2D3 also reduces the production of IL-1α, IL-1β, and IL-6 [43]. In monocytes isolated from RA patients and stimulated with lipopolysaccharide (LPS), the addition of 1,25(OH)2D3 and dexamethasone induces the development of a tolerogenic subset of dendritic cells (tolDC), with a stable anti-inflammatory phenotype. These cells are able to suppress the immunogenic activity of mature DC, inhibiting T cells proliferation and cytokines production [44]; more specifically, tolDC suppress the development of Th17, and act simultaneously to ameliorate the severity and progression of arthritis in a murine model [45]. In a recent study, DBA1/J mice induced for CIA were treated intraperitoneally with 1,25(OH)2D3 which drove the CD4+ differentiation from a Th1-Th17 to a more favorable Treg phenotype. This was associated to decreased incidence and lower clinical scores of arthritis, and reduced erosive burden [46].

Fibroblast-like synoviocytes (FLS) are another interesting target of vitamin D activity. These cells are not mere innocent bystanders in RA; they actively participate in the inflammatory response, specifically contributing to the development of bone erosions, one of the most important pathogenetic moments in the course of the disease. 1,25(OH)2D3 acts on FLS isolated from RA patients, significantly reducing the number of invasive FLS in vitro; this was associated to a reduction of cytoskeleton reorganization and to a suppression of metalloproteinases (MMPs) production [47]. Moreover, 1,25(OH)2D3 enhances FLS apoptosis preventing RA progression [48,49]. In line with the previous findings, Wen et al. reported that 1,25(OH)2D3 is able to suppress the IL-22 induced synthesis of RANKL by FLS; finally, 1,25(OH)2D3 inhibits the in vitro osteoclastogenesis [50].

To summarize the currently available preclinical data, we can state that there is quite strong evidence that the active form of vitamin D is able to prevent the development of murine models of chronic arthritis; furthermore, it is promising as a therapeutic tool. In this setting, 1,25(OH)2D3 mainly drives immune response by acting on APC and creating a more tolerogenic and less inflammatory phenotype and directly suppresses synovial proliferation and joint erosions.

However, how these convincing evidences translate to clinical practice is less clear. Should we really postulate a potential protective role for vitamin D in vivo and, therefore, is hypovitaminosis D a risk factor for RA? And again: can we really hypothesize for vitamin D a role in the treatment of RA?

Concerning the first question, the literature is controversial. Hypovitaminosis D is undoubtedly highly prevalent in patients affected by RA [51], however, as shown by a systematic review on this topic, while some authors reported a statistically lower vitamin D plasma concentration in RA than in the general population, this observation was not replicated by others [52]. We should always keep in mind that what we measure to evaluate vitamin D status is 25(OH)D3 which is a more stable intermediate metabolite, but which does not automatically reflect the real activity of vitamin D on immune cells, being influenced by the degree of activation mediated by CYP27B1 and inactivation mediated by CYP24A1. Furthermore, the immunological mechanisms triggering RA date back years from disease onset; therefore, the evaluation of vitamin D status in a patient with an already established disease is not informative for the potential protective role of this hormone on the pathogenesis of RA. It is interesting to note that data from the Iowa Women’s Health Study (IWHS) suggest a potential protective effect of higher vitamin D intake on incident RA in a very large prospective cohort [53]. While it is unclear whether RA patients are more prone to hypovitaminosis D, there is convincing evidence that vitamin D status inversely correlates with disease activity when the disease is established [54,55].

It seems therefore reasonable to postulate a potential role for vitamin D for the treatment of RA [56,57]. We recently reviewed the current evidences supporting the use of cholecalciferol as a disease modifying antirheumatic drug in RA [58]. As discussed in the paper, the literature about this topic is controversial because of the small sample size of the interventional studies and their wide heterogeneity, both in terms of endpoints used to measure the outcome and supplementation regimens considered. Nevertheless, we managed to draw the following conclusions:

1. Hypovitaminosis D might contribute to the neuropathic pain, often present in RA [59]; the correction of vitamin D status, even with low doses aiming to normalize plasma levels, is convincingly effective in ameliorating pain [60];
2. The effect of cholecalciferol supplementation on disease activity is controversial; however, when vitamin D was used at higher doses, the supplementation regimen was generally beneficial [61,62]. This suggests that, possibly, the effect on immune system of vitamin D requires higher plasma levels than those necessary for bone health. Indeed, plasma 25(OH)D3 approximately doubles the synovial concentration [63]; the anti-inflammatory properties of this intermediate have been demonstrated at a 50-100 nM concentration. Thus, the plasma level required for bone health is probably ineffective to elicit immune regulation [37].
3. Vitamin D status correction is still to be considered quintessential for bone health, particularly in RA patients who are prone to bone loss, although the preferable regimen is not defined yet [64,65].

In conclusion, clinical studies are less convincing than preclinical data but, again, we should consider that the supplementation studies have been mainly conducted with an intermediate metabolite (cholecalciferol), which requires activation, while the in vitro studies have been performed with the active 1,25(OH)2D3. This might justify these discrepancies, at least in part.

### 2.2. Spondyloarthritis

Vitamin D status is generally poor in patients with spondyloarthritis (SpA) [66]. Despite the fact that vitamin D derivatives are topically employed in cutaneous psoriasis, its potential role in the pathogenesis and management of Psoriatic Arthritis (PsA) is less investigated than in RA. Different studies reported lower 25(OH)D3 plasma concentrations in PsA patients than in healthy controls [67], being vitamin D status inversely associated with inflammatory markers [68]. Data on effects of cholecalciferol supplementation on disease course are scarce. In 1990, Huckins and colleagues reported that the daily administration of calcitriol, in a small cohort of ten PsA patients, significantly improved tender joints count [69]. A further report belonging to 2009 showed the potential immunological effects of alphacalcidol administration, which was associated to a persistent improvement of disease activity [70]. Clearly, controlled trials are lacking and further studies are required to elucidate the potential therapeutic use of vitamin D in PsA.

In ankylosing spondylitis (AS) data are even less conclusive. The current evidences deriving from systematic reviews and meta-analysis suggest that AS patients harbor lower vitamin D values than controls [71,72]. However, whether an association links vitamin D status to disease activity is debated, with some papers demonstrating an inverse association between 25(OH)D3 and disease activity [73,74] and others refusing this finding [75,76]. Currently, there are no trials investigating the effect of vitamin D supplementation on disease activity. 

## 3. Vitamin D and Autoimmune Connective Tissue Diseases

### 3.1. Systemic Lupus Erythematosus 

Systemic lupus erythematosus (SLE) is a chronic multisystemic autoimmune disease characterized by tissue and organ inflammation and damage in relation to the production of autoantibodies directed against nuclear antigens.

In animal models of SLE, the relationship between vitamin D and disease manifestations is controversial, suggesting that the effect of vitamin D may be different depending on the target organ, explaining the clinical observation of different responses according to disease phenotype. In MRL/1 SLE mice, 25(OH)D3 reduced cutaneous lesions, proteinuria, and anti-double strand DNA (anti-dsDNA) autoantibodies [77], while cholecalciferol was associated with the worsening of histopathological damage in the NZB/W mice [78]. Recently, vitamin D supplementation in a rat model of SLE ameliorated their cognitive function by reducing the process of apoptosis in the hippocampus [79]. In a model of pristane-induced SLE in female BALB/c mice, supplementation with vitamin D improved arthritis, but not renal injury [80]. 

A reduction in anti-dsDNA production was observed when SLE-derived peripheral blood mononuclear cells were incubated with calcitriol [81]. In vitro, vitamin D reduced the state of activation of APC from SLE patients, inhibiting the expression of CD40, MHC class II, and CD86 molecules [82]. Likewise, treatment with vitamin D reduced the activation of DCs and the expression of genes related to IFN-alpha [83]. Vitamin D supplementation demonstrated a beneficial effect on B and T cell homeostasis derangement during the course of SLE as shown in two independent cohorts, by increasing Treg e Th2 [84] and decreasing Th17 and Th1 cells, and memory B cells [85]. 

Vitamin D deficiency has been observed to be associated with SLE disease expression, relapses, and pathogenesis [86,87]. Systematic reviews and meta-analyses have been published on the significance of lower circulating levels of vitamin D in patients with SLE of different ethnicity when compared to healthy controls [88,89,90]. In particular, vitamin D status inadequacy was more prevalent among unsupplemented SLE patients living at a latitude beyond the 37° parallel north and [88]. The reduced sun exposure due to photosensitivity and the use of photo-protection, as well as the alteration of its renal metabolism, the presence of VDR polymorphisms which reduce the cell responsiveness to the hormone or the genetic variants of two genes encoding key enzyme regulators of endogenous production, were all described as additional risk factors for vitamin D insufficiency in SLE patients as compared to healthy controls [91,92]. Medications used for the treatment of SLE may also influence the vitamin D status. Data on hydroxychloroquine (HCQ) are still controversial: some authors found lower vitamin D levels in patients treated with HCQ [93], although others found opposite results or did not observe any association [94,95]. Noticeably, chronic corticosteroid use reduces intestinal absorption and accelerates the catabolism of 25(OH)D3 and 1,25(OH)2D3 through an increase in CYP24A1 activity [96,97]. 

Active SLE patients with lupus nephritis harbored significantly lower vitamin D levels than their counterparts [98]. Furthermore, vitamin D exerts a protective role in podocyte injury induced by autoantibodies from patients with nephritis [99]. Lower vitamin D levels in the bloodstream appear to be associated with worse disease activity, as well as with extra-musculoskeletal complications such as fatigue [100], cardiovascular risk [101], and cognitive impairment [102,103]. In addition, a recent longitudinal cohort analysis showed that vitamin D deficiency was associated with more active disease at baseline and over time, as well as a trend toward more severe lupus flares [104].

Despite the debate about low levels of vitamin D being either the cause or the consequence of SLE [105], vitamin D supplementation should be deemed as integral part of SLE management strategies [88].

Concerning the relationship between vitamin D supplementation and SLE disease activity, two RCTs [106,107], one open clinical trial [108] and a cohort study [109] found that vitamin D supplementation is able to reduce disease SLE activity, while two cohort studies [110,111] and a RCT [112] failed to observe any significant variation. Schedules and dosages were highly variable across these studies. SLE serology does not seem to be affected by vitamin D supplementation given both with an intensive or a standard regimen [110], while a higher vitamin D dose was able to reduce anti-dsDNA antibodies [85]. Some authors demonstrated that vitamin D supplementation may play a role in decreasing urine protein-to-creatinine ratio and the likelihood of clinical proteinuria [109]. Table 1 reports the major finding of prospective studies of vitamin D supplementation in SLE patients.

Osteoporosis and fractures greatly contribute to bone damage in SLE patients, symptomatic fractures being reported in 6–42% of patients following SLE diagnosis [113]. Vitamin D deficiency is considered as a major risk factor for bone damage along with persistent activity of disease, use of glucocorticoids, kidney disorders, premature menopause, and physical inactivity that is due to chronic pain and fatigue. Vitamin D supplementation is indicated for both prevention and treatment of osteoporosis in SLE patients at the daily oral dose of 800–2000 UI of cholecalciferol so as to maintain serum vitamin D levels above the target of 30 ng/ml [114]. Of note, cardiovascular events are the major comorbidities in SLE patients being accelerated atherosclerosis responsible for their premature cardiovascular diseases [115], and a recent study suggested vitamin D and calcium supplementation may have effects on the arterial stiffness of SLE patients. Longitudinal studies are indeed warranted on larger affected populations with longer follow-ups [116].

Interventional studies also pinpointed the relevance of vitamin D supplementation safety. Vitamin D toxicity is possible, although rare, and the main complications are hypercalcemia and hypercalciuria. Globally, the dosages used in these studies appeared to be safe and none of these studies described an increased occurrence of lithiasis. 

In conclusion, vitamin D supplementation is strongly recommended in SLE patients. Firstly, for the prevention of glucocorticoid induced osteoporosis, but also for possible immunomodulatory effects that remain to be fully elucidated [117]. Current vitamin D supplementation strategies are not sufficient in rising serum levels of vitamin D in every patient, therefore a treat-to-target approach could be a possible solution. For this reason, an initial measurement of serum levels of vitamin D should be done for each patient. As a general rule, 100 IU/day of vitamin D intake is needed to increase 1 ng/mL of serum 25(OH)D, which takes about 3 months to became stable once supplementation is started [118].

### 3.2. Antiphospholipid Syndrome

The antiphospholipid syndrome (APS) is a systemic autoimmune disease characterized by thrombotic manifestations and/or pregnancy-related complications in patients with confirmed antiphospholipid antibodies (aPL). Lower vitamin D levels have been described in APS patients when compared to healthy controls [119,120,121], particularly in patients with thrombotic disease [122]. However, no stringent lifestyle recommendations or dietary restrictions have been expressed for patients with APS with reference with vitamin D deficiency [123]. Studies in vitro showed that, in monocytes stimulated by anti-b2glycoprotein I (anti-b2GPI) antibodies derived from APS patients, vitamin D can inhibit the expression of tissue factor [124]. The relationship between vitamin D and thrombosis has been further investigated [125,126], as VDR activators can intervene to control the expression of several thrombogenic factors [127]. Nevertheless, 25(OH)D3 supplementation in obese women failed to improve haemostatic parameters as assessed by calibrated automated thrombogram [128]. Vitamin D deficiency was found as a risk factor also for recurrent pregnancy losses in patients with aPL, in association with other signs of autoimmunity [129]. In early pregnancy, vitamin D is produced by trophoblasts contributing to decidualization for successful pregnancy [130,131]. According to the EULAR recommendations, supplementation with calcium, vitamin D, and folic acid should be offered to patients with APS (and/or SLE), particularly in the case of vitamin D deficiency in the first trimester of gestation and in patients receiving glucocorticoids and/or heparin for their detrimental effects on bone mass [132].

## 4. Vitamin D and Autoimmune Endocrine Diseases

### 4.1. Type 1 Diabetes Mellitus

Type 1 diabetes (T1DM) is an autoimmune disease characterized by an immune-mediated destruction of pancreatic beta-cells, which leads to a lifetime dependence on exogenous insulin [133,134]. The causes of T1DM onset are incompletely defined and a combined effect of genetic predisposition and environmental triggers has been hypothesized [135,136]. It is estimated that 542,000 children aged under 14 years are affected by T1DM, with an incidence that has increased by 3-4% over the last thirty years [137]. The pathogenetic role of vitamin D in T1DM onset is debated. The Type 1 Diabetes Genetic Consortium, which genotypically characterized 38 single nucleotide polymorphisms (SNPs) in more than 1500 families with T1DM, did not observe correlations between SNPs in the VDR and T1DM [138], even if associations between some VDR polymorphisms (BsmI, FokI, TaqI, ApaI) and T1DM have been pinpointed in several population studies [139,140,141,142,143,144,145,146,147,148]. In 2017, Sahin OA et al. [149] published a meta-analysis, with the aim to evaluate the relationship between ApaI, BsmI, FokI, and TaqI polymorphisms of VDR and T1DM in children. The authors analyzed 9 studies, including a total of 2070 patients and controls. Their results showed that BsmIBb, BsmIBB, and TaqItt polymorphisms were linked to a higher risk of T1DM onset, whilst BsmIbb and TaqITT seem to play a protective role [149]. 

In preclinical studies, an intriguing role for vitamin D in the pathogenesis of T1DM has been postulated. Vitamin D prevents β-cell apoptosis caused by cytokine exposure and restores insulin secretion [150]. In vitro and animal studies suggested that vitamin D could exert a modulatory effect on the immune system by showing that supplementary treatment with vitamin D may have a protective role against the T1DM onset in experimental models [151]. In humans, the prevalence of T1DM is associated with ultraviolet B radiation, altitude and latitude as well as it displays a seasonal pattern, suggesting a pathogenetic role for vitamin D in this context [152,153,154]. Moreover, lack of vitamin D supplementary treatment in childhood and lower maternal 25(OH)D3 plasma levels during pregnancy could be related to an increased risk of T1DM onset later in life [155,156]. Furthermore, a meta-analysis including five observational studies reported that vitamin D supplementary treatment during infancy is associated with a lower risk of T1DM [151]. Finally, other studies and meta-analyses demonstrated that patients with T1DM harbor lower serum levels of vitamin D compared to controls and showed how vitamin D deficiency could be associated with a worse glycaemic control [157,158,159,160,161,162,163,164,165]. However, two large population-based Danish studies did not observe any association between vitamin D concentrations and later risk of developing T1DM [166], while other studies did not find significant differences in vitamin D levels between patients with T1DM and controls [167,168,169]. 

The results of interventional studies are also conflicting. An open-label randomized trial on 70 T1DM patients, treated with low dose of calcitriol or nicotinamide for one year, did not observe differences in C-peptide or HbA1c levels, while a modest effect of calcitriol on the residual pancreatic beta-cell function was observed [170]. In 2013, Papadimitriou et al. demonstrated that 0.25 μg/day of calcitriol administered to 12 high-risk children with T1DM was able to abolish the presence for anti-GAD65 antibodies and insulin autoantibodies after 6 months [171]. Moreover, the supplementary treatment with calcitriol in subjects with latent autoimmune diabetes in adults (LADA) seems to exert a protective effect on residual pancreatic beta-cell function compared with patients treated only with insulin analogue [172]. On the contrary, Walter et al. observed that 0.25 mg per day of calcitriol did not seem to preserve beta-cell function [173]. According to a later systematic review of 7 randomized controlled clinical trials, supplementary treatment with vitamin D, in particular with alpha-calcidol and cholecalciferol, might attenuate the natural course of the T1DM [174]. Finally, vitamin D supplementation seems to reduce glycaemic variability, insulin requirements and hypoglycaemia rates [175].

In conclusion, these data suggest that an adequate supplementary treatment with vitamin D could be able to improve glycemic control in T1DM patients and to prevent the disease onset in high risk subjects.

### 4.2. Thyroid Autoimmunity

Autoimmune thyroid disorders (AITD) are the most common autoimmune diseases with a prevalence of 5% in the general population and an increasing trend in the incidence over the years [176]. The aetiology of AITD is multifactorial, involving genetic predisposition and environmental factors [177]. Hashimoto’s thyroiditis (HT), and Graves’ disease (GD) represent the most frequent AITD, immunologically characterized by circulating antithyroid antibodies and thyroid glandular infiltration of lymphocytes, respectively [178].

**Figure 2:**
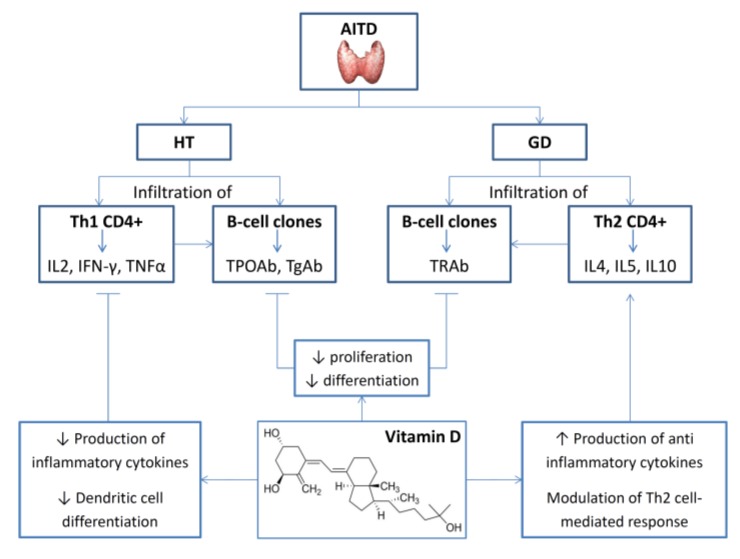
Hashimoto’s thyroiditis (HT) is a T-cell-mediated endocrine autoimmune disease. Patients harbor higher thyroid peroxidase antibodies (TPOAb) and TgAb serum levels and thyroid intraglandular infiltration of B and T lymphocytes with CD4+ Th1 subtype predominance. Graves’ disease (GD) is characterized by a prominent Th2-mediated humoral response, which induce the expression of stimulatory antibodies. Vitamin D is able to reduce the proliferation and differentiation of B cells into plasma cells and induce the apoptotic cascade of immunoglobulin. In this context, vitamin D inhibits the Th1 cells proliferation as well as the Th1-mediated cytokines production (IL-2, IFN-γ, and TNFα) and modulates Th2 cells and cytokines production (IL-4, IL-5, and IL-10) inducing Th2 phenotype. Arrows are used to illustrate decreased (↓) or increased (↑) production of specific actions, cells or molecules.

Several preclinical and clinical studies observed a relationship between hypovitaminosis D and AITD [179,180]. Figure 2 illustrates the main pathophysiological correlates of vitamin D effects on AITD. 

In a 1990 study based on evidence that in vitro inhibition by cyclosporin A (CsA) is potentiated by calcitriol, Fournier et al. investigated in vivo the influence of both molecules by using an experimental mouse model of AITD [181]. The study demonstrated a synergistic role of calcitriol in reducing the incidence of thyroid autoimmunity and the severity of histological lesions. Several years later, Borgogni et al. investigated the effects of elocalcitol, compared with methimazole, on CXCL10 secretion induced by proinflammatory cytokines in human thyrocytes. Their results showed that elocalcitol inhibited IFN-γ and TNFα-induced CXCL10 protein secretion more effectively than methimazole and promoted a shift toward a Th2 response [182].

Animal models deploying an immunization protocol with thyroid stimulating hormone receptor antibody (TSHR Ab) showed that vitamin D deficiency promoted persistent hyperthyroidism, leading to speculate on a potential modulatory effect of vitamin D on thyroid function [183]. One year later, Liu S et al. analyzed the effect of calcitriol on thyroid inflammation and Th1/Th2 cells in mice with experimental autoimmune thyroiditis [184]. The authors demonstrated that calcitriol can help to maintain normal autoantibodies and citokines levels as well as the thyroid glandular structure when administered before the onset of the experimental damage.

The mechanisms behind the putative beneficial effects of vitamin D on AITD mentioned above are unclear, but they are possibly related to its known immunomodulatory and anti-inflammatory properties.

Hashimoto’s thyroiditis (HT) is often characterized by hypothyroidism and the production of thyroid autoantibodies like thyroid peroxidase antibodies (TPOAb) and thyroglobulin antibodies (TgAb), and with thyroid glandular lymphocytic infiltration [185].

Several observational and interventional studies found a potential link between hypovitaminosis D and a higher risk of HT onset. The first observational study was published by Goswami et al. in 2009 and observed an inverse correlation between serum vitamin D levels and TPOAb titres [186]. Later, studies confirmed this negative association [187,188,189,190,191,192] and also reported a negative correlation between vitamin D and TgAb serum concentrations [189,191]. Several studies demonstrated that TPOAb positivity was more frequent in subjects with hypovitaminosis D compared to subjects with sufficient hormone levels [187,193,194,195,196,197,198,199]. Further studies also demonstrated an association between the VDR polymorphisms and a higher incidence of HT, particularly VDR rs731236, rs1544410, and rs2228570 [200].

Human intervention studies demonstrated that cholecalciferol supplementation was associated with an important decrease in TPOAb and TgAb levels both in patients with vitamin D sufficiency and deficiency [201,202,203,204]. In a recent 3-month RCT on women with HT, Chahardoli R et al. not only observed a significant decrease of TgAb after cholecalciferol supplementation (50.000 U), but also reported a significant reduction of TSH levels [205].

Graves’ disease (GD) is a common autoimmune disease with an incidence of 14/100,000 per year and is characterized by the presence of thyroid stimulating hormone (TSH) receptor autoantibodies which cause hyperthyroidism, goiter, and ophthalmopathy [206,207]. Although some studies observed an increased prevalence of GD in subjects with hypovitaminosis D, the association between these two conditions is not so straightforward [208]. The first observational study on this topic examined vitamin D levels in female population with and without remission of GD [209]. The authors found higher vitamin D in GD women with remission as compared to those without. One year later, Unal et al. showed that patients with GD harbor lower vitamin D levels than normal controls [189]. These results have been further confirmed by three recent observational studies [197,210,211]. Moreover, Xu and colleagues conducted a meta-analysis with 26 clinical studies, showing that hypovitaminosis D seems to double the risk of GD onset [212]. Recently, an interventional study was designed to evaluate whether daily supplementary treatment with vitamin D reduces GD recurrence. The results showed that GD recurrence occurred earlier in patients not receiving vitamin D supplementation [213]. Finally, as in the case of HT, polymorphisms of VDR gene seem to be related to a higher risk of GD occurrence in several investigations, but with a low statistical power [214].

Summarizing, studies observed a correlation between hypovitaminosis D and thyroid autoimmune diseases. Cholecalciferol supplementation seems to exert beneficial effects on thyroid autoimmunity. However, large multicenter studies are needed to determine the impact of supplementary treatment with vitamin D on clinical outcomes in AITD. 

### 4.3. Addison’s Disease

Addison’s disease (AD) represents a rare adrenal autoimmune disease characterized by a current prevalence of 100–140 cases per million population [215]. A steady increase of the prevalence of AD has been observed over the years, particularly in women [216]. AD clinically occurs with adrenal insufficiency, caused by an autoimmune-mediated destruction of adrenal cortex [217]. Although the pathogenesis of AD has not been completely elucidated, an interplay role between HLA haplotypes and environmental factors has been postulated [218,219]. In addition, different genes responsible for vitamin D metabolism and VDR gene polymorphisms are implicated in AD onset [220,221,222,223]. Only few studies evaluated the potential relationship between AD and circulating vitamin D levels. In 2013, Ramagopalan and colleagues investigated the potential role of hypovitaminosis D in influencing the pathogenesis of immune-mediated diseases [224]. The authors observed that in patients with hypovitaminosis D there were significantly higher rates of AD and other autoimmune diseases. Subsequently, Pazderska et al. demonstrated that subjects born in the winter had a higher risk of AD occurrence [225]. These results suggested that hypovitaminosis D, coupled with exposure to seasonal viral infections, could deregulate the innate immunity increasing the risk of AD onset [225]. In 2018, Penna-Martinez and colleagues conducted a pilot trial to investigate the effects on the immune response of patients with AD of high dose cholecalciferol therapy (4000 IU/day) over a 3-month period. The authors observed that supplementation with cholecalciferol could interfere with the late activation of monocytes and T-cells in subjects with AD, providing novel insights about immunomodulation in AD [226].

It may thus be concluded that only preliminary evidence exists to suggest that hypovitaminosis D and AD could be closely related, considering the potential role of vitamin D in modulating the immune response.

### 4.4. Autoimmune Polyendocrine Syndromes

Autoimmune polyendocrine syndromes (APS) represent rare conditions characterized by autoimmune activity against multiple endocrine organs [227]. The two major APS, APS-1 (AD, hypoparathyroidism and candidiasis), and APS-2 (AD, AITD and T1DM), have Addison’s disease as a prominent component. APSs include APS-3 (AITD and other autoimmune diseases) and APS-4 (autoimmune polyendocrinopathies that do not fulfil the criteria of APS 1 to 3) [228]. Across different countries, the estimated prevalence of APS is reportedly around 1:80,000 [229]. To date, only a single clinical study evaluated the potential association between circulating vitamin D levels and APS. The authors observed that subjects with APS showed lower 25-OHD serum concentrations compared to healthy controls [230]. It is currently unclear if hypovitaminosis D could represent a cause of APS onset rather than a consequence.

## 5. Vitamin D and Autoimmune Liver Diseases

Autoimmune liver diseases, despite being relatively rare, represent a relevant cause of liver-related morbidity and mortality. Autoimmune hepatitis (AIH) is an immune-mediated chronic liver disease with no defined cause, accounting for about 16–18 cases per 100,000 inhabitants in Europe. The main target of auto-inflammation are primarily hepatocytes. AIH can range from a mild, asymptomatic disease to a severe form of acute or even fulminant hepatitis and appears to be more frequent in women, although its incidence among men is on the rise [231].

Cholestatic autoimmune liver diseases include primary biliary cholangitis (PBC), which is a chronic small bile duct cholangitis affecting about 1 in 1000 women aged over 40 worldwide and presents with non-suppurative granulomas [232,233], and primary sclerosing cholangitis (PSC), affecting mainly young men with both small and large bile ducts involvement [234]. Although the complex pathogenesis of autoimmune liver diseases is still incompletely understood, it has been hypothesized a role for vitamin in this setting. Many studies have investigated the influence of 1,25(OH)2D3 in the liver. It is known that it directly influences cytoplasmic calcium levels in rat hepatocytes [235] and promotes liver regeneration after hepatectomy in murine models [236]. It has been found that vitamin D increases the expression of P450 cytochromes (CYP3A4, CYP2D6, CYP2C9) in human primary cultured hepatocytes [237]. In addition, it has been reported that the isoenzyme CYP2D6 could potentially convert vitamin D3 into 25-hydroxy-vitamin D [238]. CYPs are common targets of immune-mediated reactions in autoimmune liver diseases. In particular, CYP2D6 expressed on the membrane of hepatocytes is the major auto-antigen for anti-liver kidney microsome type 1 (LKM1) antibodies in type 2 AIH [239]. The clinical relevance of anti-CYP2D6 auto-reactivity in autoimmune hepatitis is supported by animal models: mice infection with CYP2D6-expressing Ad5 adenovirus leads to the production of anti-CYP2D6 IgG that cause immune-mediated liver injury by recognizing the same epitopes targeted by human auto-antibodies [240].

There are several potential implications of vitamin D in AIH, due to both genomic and non-genomic functions, such as suppression of MCH-II antigen expression and increase of CTLA-4 production [241]. As shown in a model of Concanavalin A (Con-A) induced autoimmune hepatitis in mice [242], calcitriol decreases serum ALT levels, attenuates histological liver damage, and decreases IFN-γ levels.

The liver immunomodulatory effect of vitamin D appears to be related to the 25-hydroxylation, which creates a negative feedback for local inflammation and leads to Th2 polarization [243], due to the inhibition of pro-inflammatory TNF-α, IL-2, IL-12, IL-17, IFN-γ, and promotion of IL-4, IL-5, and IL-10 production [244]. In addition, calcitriol may exert an anti-oxidant by increasing intracellular glutathione and counteracting reactive oxygen species, which have been suggested to be involved in AIH and PBC. In fact, a vitamin-D poor diet has been shown to increase NADPH in rat hepatocytes [245]. Moreover, a putative immunomodulatory role of vitamin D seems to be linked to its influence in hepatic invariant natural killer T-lymphocytes (iNKT) development. These are mostly found in hepatic sinusoids and are able to activate hepatic stellate cells, as well as to mediate hepatocytes’ killing [246]. Liver iNKT cells constitutively express OX40, which is involved in inducing proinflammatory and profibrotic liver injury, as reported by Lan and colleagues [247]. Although iNKT role in autoimmune liver disease is still not fully understood, it has been reported that these activated cells mediate a major role in inflammation and hepatocyte death in a murine model of Con-A-induced autoimmune hepatitis [248].

In this setting, animal models have shown that adequate levels of vitamin D are essential for normal iNKT development. It seems that vitamin D accounts for the number of iNKT cells, whereas the VDR plays a role for both iNKT number and function [249]. Interaction of vitamin D with its receptor VDR results in many effects other than its role in T cell differentiation. This receptor is expressed by CD4+ T lymphocytes, CD8+ T lymphocytes, B lymphocytes, DCs, NK cells and macrophages [250]. VDR receptor is widely expressed at low levels within hepatocytes, whereas high levels of VDR expression have been demonstrated in hepatic non parenchymal cells, such as biliary epithelial cells and hepatic stellate cells (HSCs) [251]. VDR-mediated signaling in HSCs seems to act by antagonizing the potent hepatic profibrogenic TGF-β/SMAD-dependent transcriptional pathway [252].

The locally produced 1,25(OH)2D3 is also responsible for a negative feedback for the expression of VDR in bile duct cells [243]. VDR could be involved in maintaining bile duct integrity, suggesting a potential involvement in cholestatic liver damage. Firrincieli et al. found that VDR knockout mice presented an impairment in bile acid homeostasis, ductal reaction, as well as disruptions in biliary epithelial junctions following a biliary-type liver injury [253]. Moreover, the signaling activated by vitamin D-VDR interaction can modulate the transcriptional response related to bile acid resulting in a putative protection of hepatocytes from cholestatic injuries [254].

An additional anti-inflammatory action of 1,25(OH) is related to the fact that it increases the activity of MPK-1, consequently inactivating the mitogen-activated protein kinases (MAPKs); the subsequent modulation of nuclear factor of activated T-cells (NFAT), NF-κB, and nuclear histone deacetylase, reduces inflammatory activity in immune-mediated diseases [244].

Vitamin D also increases the glucocorticoid anti-inflammatory function by stimulating the synthesis of mediator complex subunit 14 (MED14); it induces the binding of the activated glucocorticoid receptor to the glucocorticoid-responsive element in the MPK-1 gene promoter. In addition, 1,25(OH)2D3 emphasizes the negative effect of glucocorticoids on the production of LPS-induced IL-6 [255] and interferes with the translocation of NF-κB to the nucleus [256]. As glucocorticoids are the main therapeutic agents used for autoimmune hepatitis, this finding might have potentially therapeutic implications for a putative role of vitamin D supplementation to booster steroidal treatment efficacy.

Calcitriol has antifibrotic and anti-proliferative effect on the liver, as shown both in in vitro and in vivo models [257]. Liver damage and inflammation frequently result in hepatic fibrosis. Pro-inflammatory cytokines activate HSCs, which transform into myofibroblasts with consequent proliferation, contractility, loss of intracellular retinoid stores, cytokine production, and extracellular matrix deposition [258]. It has resulted that 1,25(OH)2D3 inhibits proliferation, activation, and transformation of HSCs into myofibroblasts in a murine model of live injury induced by thioacetamide [259].

Emerging evidence suggests that vitamin D could protect against liver fibrogenesis, due to different mechanisms. One of the most important is the signaling mediated by VDR [257] which, as discussed before, is extensively expressed on HSCs: Ding et al. showed that VDR knockout mice develop spontaneous liver fibrosis [252], because of an impairment in VDR/SMAD genomic feedback.

It has been shown that 1,25(OH)2D3 decreased the mRNA transcription for type 1 collagen in cultured human HSCs by interacting with VDR [260]. Vitamin D, besides repressing collagen I and III expression, strengthens the expression of matrix metalloproteinase-8 (MMP-8), a collagen cleaving enzyme [261].

Moreover, activated HSCs secrete connective tissue growth factor (CTGF) as well as other matricellular proteins [262]. In a rat model, a significant reduction of CTGF expression has been observed after vitamin D treatment [263]. Similar results were obtained by evaluating the correlation between circulating levels of CTGF and severity of liver fibrosis in a cohort of patients with biliary atresia [264].

Supplementation of vitamin D may prevent liver fibrosis development, as resulted in murine models of primary sclerosing cholangitis [265] and thioacetamide-induced cirrhosis [259].

It is widely reported the higher frequency of vitamin D insufficiency or deficiency among autoimmune liver diseases patients compared to healthy subject. Scarcity of vitamin D has been evaluated as a prognostic marker, linked to disease severity [266,267,268]. Serum levels of 1,25(OH)2D3 lower than 20 ng/mL have been described in a large proportion of patients with non-cholestatic autoimmune liver diseases (51–92%), and vitamin D insufficiency has been reported in 17–23% of patients [244].

There are several studies focusing on the potential relation of low vitamin D level and chronic liver disease, irrespective of the cause, as reviewed by Chen et al. [243]. Stokes et al. have found low vitamin D concentration to be independent predictor of mortality in cirrhotic patients (OR 6.3; 95% CI 1.2–31.2, P = 0.012): they also reported an inverse correlation between serum levels of vitamin D and the stage of cirrhosis (Child–Pugh score and model of end-stage liver disease–MELD score), mainly associated with liver failure and infections [269]. Despite that vitamin D deficiency has not resulted as a predictor of poor prognosis in AIH by itself, it has been proposed as a biomarker with potential prognostic role in this setting, as it resulted associated to more severe interface hepatitis and worse fibrosis scores; the lower the vitamin D levels, the higher appeared to be the rate of a poor response to treatment [270]. Comparable results were found by Ebadi et al. [266].

Moreover, it has been reported that 25(OH)D3 level increases in patients with AIH who are responsive to classic steroid therapy, while it does not rise in non-responders [270].

A potential prognostic value of vitamin D levels has also been described in PBC. Agmon-Levin et al., in fact, have shown that vitamin D levels resulted significantly reduced among PBC patients compared to controls, and they correlated to higher alkaline phosphatase levels as well as advanced liver damage. Patients treated with ursodeoxycholic acid (UDCA) have been described to have higher vitamin D levels [267]. 

It has also been postulated that pre-treatment vitamin D level could be independently associated to subsequent UDCA response [271].

Regarding VDR, it has been described an association between autoimmune liver diseases and VDR polymorphisms: in particular, Vogel et al. found out that Fok1 polymorphism associated to AIH, whereas BsmI polymorphism was increased in PBC affected patients in a German population [272]. The same result has been shown in a Chinese population [273] and in a Canadian group of PBC patients, in which has been postulated that VDR polymorphisms could be an independent risk factor for a lower bone mineral density [274]. These findings suggest a putative involvement of these polymorphisms with regard to D-mediated immunomodulation, although the mechanisms remain unknown.

The implication of vitamin D-VDR signaling pathway has been studied in the PBC and PSC pathogenic process. Kempinska-Podhorodecka et al. found that in PBC livers, either cirrhotic or not, there is a significant impairment in VDR expression, resulting in enhancement of non-coding miR155 and consequent SOCS1 reduction, which probably interferes with the negative feedback on pro-inflammatory cytokines response [275]. Actually, VDR is also an important receptor for ursodeoxycholic acid (UDCA) inducing cathelicidin expression in biliary epithelial cells [243]; it also mediates many epigenetic effects, which contribute to the reduction of pro-inflammatory cytokines synthesis [276].

It should also be taken into consideration that low serum concentration of vitamin D in chronic liver disease patients might reflect a deficiency in hepatic hydroxylation due to impaired liver function [277]. The lack of proper hydroxylation may be a consequence of liver disease, but it cannot be excluded that it may pre-exist liver disease, playing a role in its pathogenesis and progression [244].

Based on what was previously discussed, we can hypothesize that 1,25(OH)2D3 may be used as a potential prognostic biomarker of disease severity and treatment response [270].

The putative therapeutic benefit provided by vitamin D supplementation in chronic liver diseases needs further study to be confirmed. A recent systematic review published in 2017 [278], including 15 trials and 1034 patients in total, concluded that no strong evidence has been found supporting the hypothesis that vitamin D supplementation confers an advantage in this setting, though none of the trials included patients with autoimmune liver disease. However, considering the synergistic cooperation of vitamin D and glucocorticoids in suppressing inflammation, it would be interesting to conduct a prospective investigation to determine whether correction of vitamin D deficiency could strengthen the effect of corticosteroids or contribute to dose reduction and individualization.

Summarizing, vitamin D, along with its relative VDR is possibly inter-related with the occurrence, treatment, and prognosis of autoimmune liver diseases and represents an interesting and intriguing topic to further explore in order to improve prevention and management of immune-mediated liver diseases.

## 6. Conclusions

Knowledge on the role of vitamin D in the control of bone health physiology has been progressively integrated by evidence that vitamin D yields pleiotropic “non-calcemic” effects in vitro and in vivo, potentially linking vitamin D status with general health. A main extra-skeletal effect of vitamin D activity is related to the immune system homeostasis. Hence, a disturbed vitamin D-VDR axis is potentially viewed as a trigger for a wide spectrum of autoimmune diseases, such as inflammatory arthritis, connective tissue diseases, endocrinopathies, and different categories of autoimmune liver diseases. In vitro and in vivo data support this link and demonstrate that, at least in experimental conditions, the modulation of vitamin D of innate and adaptive immunity can contribute to prevent the susceptibility to autoimmune diseases and improve their therapeutic management.

Unfortunately, studies cannot exclude reverse causality, i.e., that low levels of vitamin D may derive from impaired kinesis or avoidance of sunlight in people with autoimmune diseases. For these reasons, randomized controlled trials are needed on treatment with vitamin D in patients with or at risk for autoimmune diseases, so as to heighten data and accuracy on the information available on vitamin D efficiency in the clinical setting of autoimmune disorders.

## Figures and Tables

**Table 1:** Prospective studies on vitamin D effects in systemic lupus erythematosus (SLE) patients.

Author (Publication Year)	Type of Study	Number of Enrolled Patients	Type of Supplementation	Main Findings
Ruiz Irastorza et al. (2010)	Longitudinal observational	80	Cholecalciferol, 600-800 IU day p.o. (24 mos)	Improved fatigue symptoms, no correlation with SLEDAI or SDI. Side effects: not reported
Terrier et al. (2012)	Prospective	20	Cholecalciferol, 100.000 IU/wk p.o. (4 wks)	Improved naïve CD4+ T cells, regulatory T cells; reduced Th1 and Th17 cells, memory B cells, anti-DNA antibodies. No cases of hypercalcemia
Petri et al. (2013)	Prospective	1006	Ergocalciferol, 50.000 IU/wk p.o., calcium/vitamin D 200 IU/twice daily p.o.	Reduced SELENA-SLEDAI, decreased urine protein-to-creatinine ratio. Hypercalcemia rate, 0.002%
Andreoli et al. (2015)	Prospective, cross-over	34	Cholecalciferol Intensive Regimen: 300.000 IU bolus plus 50.000 IU/mo p.o. (850.000 IU/yr). Standard Regimen: 25.000 IU/mo p.o. (300.000 IU/yr) for 12 mos. Regimens switched in the second year.	No effect on disease activity and SLE serology. No cases of hypercalcemia. Slight transient hypercalciuria in 3
Piantoni et al. (2015)	Prospective, cross-over	34	Cholecalciferol Intensive Regimen: 300.000 IU bolus plus 50.000 IU/mo p.o. (850.000 IU/yr). Standard Regimen: 25.000 IU/mo p.o. (300.000 IU/yr) for 12 mos. Regimens switched in the second year.	Enhancement of T-reg cells and Th2 cytokines. No cases of hypercalcemia
Aranow et al. (2015)	Randomized, double blind, placebo controlled	57	Cholecalciferol, 2.000 or 4.000 IU/d p.o.	Well-tolerated. No effect on IFN-alpha. No cases of hypercalcemia
Lima et al. (2016)	Randomized, double blind, placebo controlled	40 (JoSLE)	Cholecalciferol, 5000 IU/wk p.o.	Decreased disease activity and improved fatigue symptoms in JoSLE patients. No cases of hypercalcemia
Rifa’i et al. (2016)	Randomized, placebo controlled	39	Cholecalciferol, 1.200 IU/d p.o.	Decreased SLE disease activity and fatigue symptoms. Side effects: not reported
Karimzadeh et al. (2017)	Randomized, double blind, placebo controlled	90	Cholecalciferol, 50.000 IU/wk p.o. for 12 wks and 50.000 IU/mo p.o. for 6 mos.	No effect on SLE disease activity. Side effects: not reported

Assessment of safety: including hypercalcemia, hyperphosphatemia or lithiasis; JoSLE: juvenile-onset SLE; IFN-alpha: alpha interferon; SLEDAI: systemic lupus erythematosus disease activity index, SDI: rheumatology damage index.
